# Ten-year follow-up of suprascapular nerve decompression in a competitive volleyball player: a case report

**DOI:** 10.1093/jscr/rjaf906

**Published:** 2025-11-14

**Authors:** Gabrielle Orie, Ellen Lutnick, Robert H Ablove, Michael Rauh

**Affiliations:** Department of Orthopaedic Surgery and Sports Medicine, University at Buffalo Jacobs School of Medicine and Biomedical Sciences, 955 Main Street, Buffalo, NY 14203, United States; Department of Orthopaedic Surgery and Sports Medicine, University at Buffalo Jacobs School of Medicine and Biomedical Sciences, 955 Main Street, Buffalo, NY 14203, United States; Department of Orthopaedic Surgery and Sports Medicine, University at Buffalo Jacobs School of Medicine and Biomedical Sciences, 955 Main Street, Buffalo, NY 14203, United States; Department of Orthopaedic Surgery and Sports Medicine, University at Buffalo Jacobs School of Medicine and Biomedical Sciences, 955 Main Street, Buffalo, NY 14203, United States

**Keywords:** suprascapular nerve injury, volleyball suprascapular nerve, open suprascapular nerve decompression

## Abstract

We present a case of 10-year follow-up in a 27-year-old female collegiate volleyball athlete with continued infraspinatus atrophy confirmed on magnetic resonance imaging (MRI) despite compliance with physical therapy and rehabilitation following open suprascapular nerve (SSN) decompression. She successfully returned to sport post-operatively; however, at 10 year follow-up, had continued weakness and pain, without evidence of nerve injury on EMG. This is the first known report of 10-year follow-up in an athlete who underwent open SSN decompression without evidence of an associated spinoglenoid ganglion cyst with persistent right infraspinatus atrophy on MRI and clinical shoulder weakness without new injury.

## Introduction

Infraspinatus atrophy in overhead activities has been associated with suprascapular nerve (SSN) neuropathy, including in volleyball players between 12.5% and 30% [1, 2]. Repetitive traction along the lateral scapular spine when the infraspinatus muscle is at maximum eccentric contraction increases the SSN distance traversed [1]. Electromyography (EMG) and nerve conduction velocity studies (NCS) assist in diagnosis [2, 3].

The etiology of infraspinatus muscle atrophy remains equivocal [2], and long-term follow-up post-SSN decompression is limited [4–7]. Most studies report 1–2 years follow-up [4, 8] describing improvements in strength but not clinical atrophy [4, 8, 9]. Age of operation [10, 11], level of decompression [11], and duration of rehabilitative therapy [10, 11] may affect recovery.

We present the first known report of 10-year follow-up in a 27-year-old female volleyball athlete with continued right infraspinatus atrophy despite compliance with rehabilitation following open SSN decompression without evidence of associated spinoglenoid cyst.

The patient agreed to publication of the associated data.

## Case report

**Figure 1:**
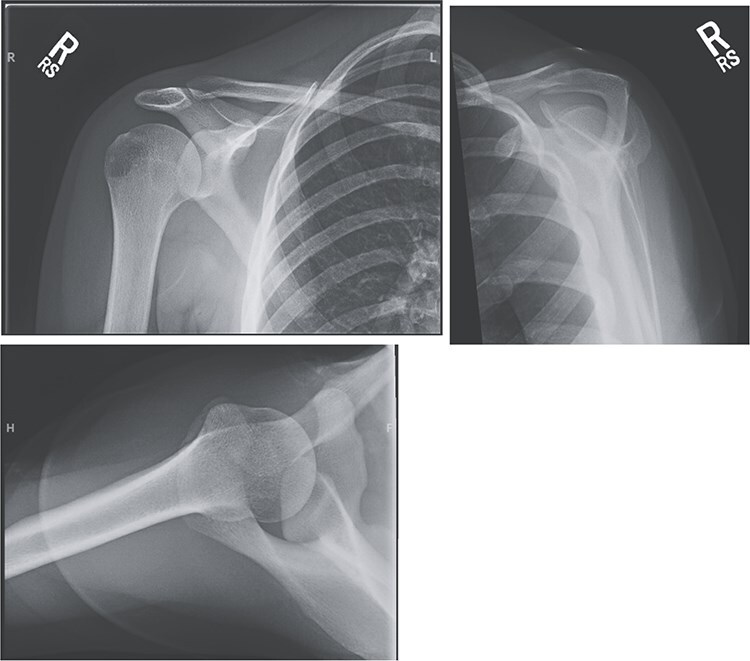
Radiographic imaging on initial presentation was without transverse scapular ligament calcification.

**Figure 2:**
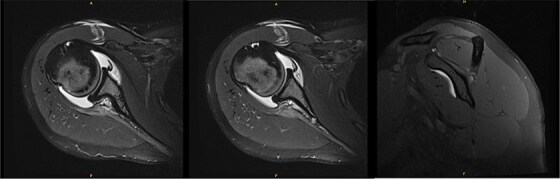
Initial MRI arthrogram revealed low-grade undersurface fraying of the supraspinatus and infraspinatus tendons consistent with internal impingement, hypertrophic posterior inferior labrum without visible tearing, and no evidence of paralabral cyst.

An 18-year-old, right-hand dominant woman presented reporting several weeks of right shoulder pain limiting participation in DIII collegiate volleyball. Examination revealed atrophy of the infraspinatus region. Muscle testing revealed weakness of infraspinatus and supraspinatus with reproducible symptoms. Radiographs were without transverse scapular ligament calcification (Fig. 1). Magnetic resonance imaging (MRI) arthrogram revealed low-grade undersurface fraying of the supraspinatus and infraspinatus tendons consistent with impingement, hypertrophic posterior inferior labrum, and no evidence of paralabral cyst (Fig. 2). Rest from hitting activities, anti-inflammatory medications, and periscapular strengthening therapy were initiated.

At 3 months, the patient had persistent pain despite compliance. NCS demonstrated absent SSN response from the infraspinatus. EMG demonstrated denervation with positive waves and fibrillation potentials, without volitional motor units, attributed to complete axonotmesis of SSN to infraspinatus, consistent with suprascapular neuropathy at the spinoglenoid notch.

Open decompression of the SSN was performed one month later from the level of the spinoglenoid notch into the two heads of the infraspinatus. The nerve was notably adhered to the posterior scapula. Following neurolysis, the SSN was freely mobile without cyst or mass, consistent with MRI.

At 15 weeks postoperatively, she demonstrated progressive deltoid and infraspinatus muscle strength without pain. She returned to collegiate volleyball with as-needed follow-up.

**Figure 3:**
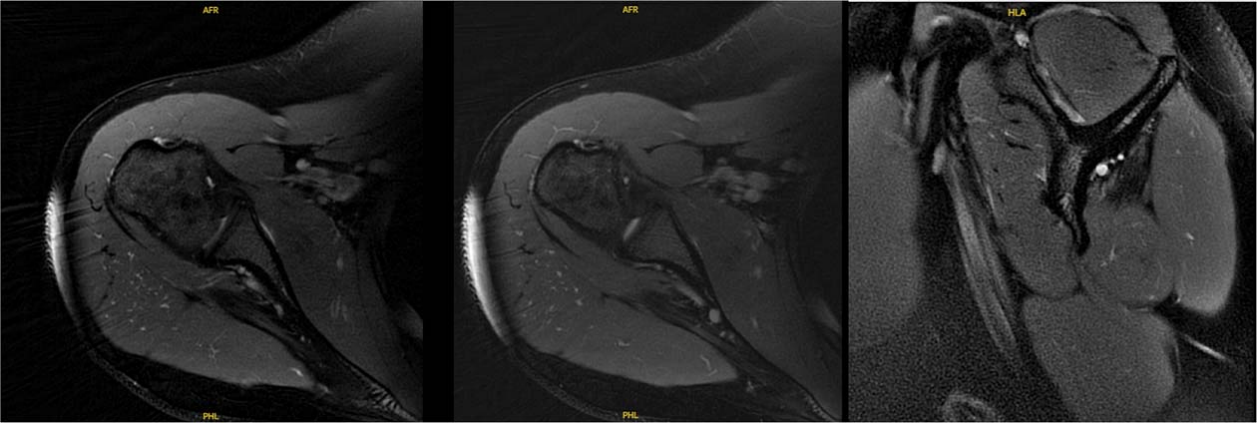
MRI 6 years after decompression revealed a diffusely atrophic infraspinatus muscle belly.

Six years following decompression, the patient presented for right shoulder pain and fatigue without new injury. Post-collegiately, she coached volleyball. MRI revealed a diffusely atrophic infraspinatus muscle belly (Fig. 3). On examination, she demonstrated persistent diminished infraspinatus, supraspinatus, and teres minor strength, pain in the Aber position, mild anterior apprehension, and a positive (+1) Sulcus sign. She proceeded with posterior capsular stretching and cuff strengthening.

**Figure 4:**
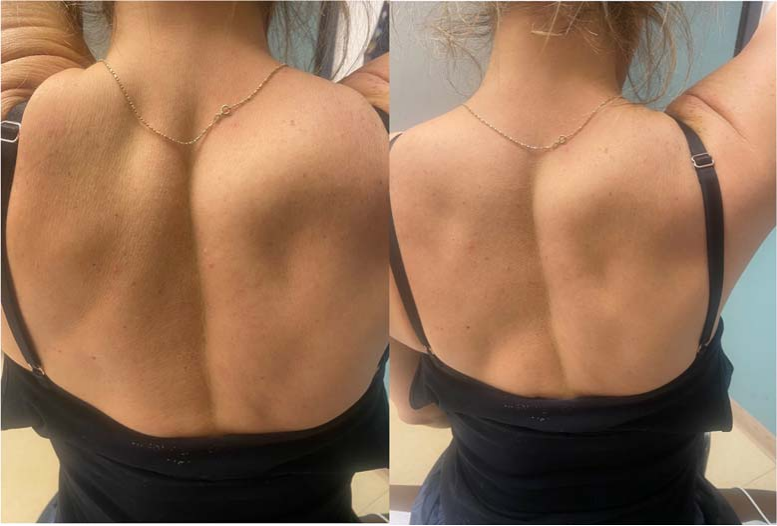
Nine and half years after decompression, the patient presented with palpable and visible atrophy of the right infraspinatus.

At 9.5 years, she presented reporting recurrent, progressive shoulder weakness and pain during external rotation during self-directed physiotherapy. There was visible atrophy of the right infraspinatus (Fig. 4). She had full symmetrical shoulder range of motion, with 4/5 strength of the right infraspinatus and a markedly weak Hornblower’s test.

**Figure 5:**
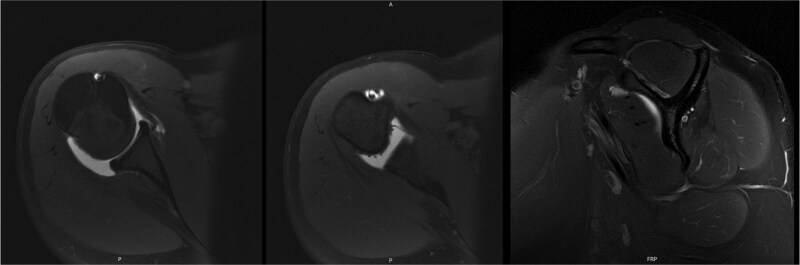
MRI at 9.5 years after decompression demonstrated increased right infraspinatus muscular tissue.

New MRI arthrogram demonstrated no evidence of new or worsening SSN compression or other pathology. Imaging demonstrated increased right infraspinatus muscular tissue (Fig. 5), however, with significant continued fatty infiltration and persistent atrophy compared to the contralateral side.

**Figure 6:**
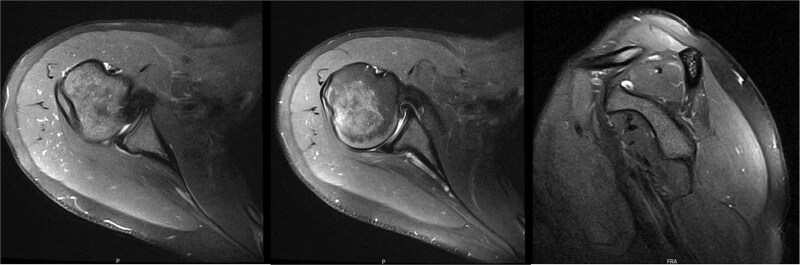
MRI at final follow-up demonstrated mild supraspinatus tendinosis, and infraspinatus atrophy with continued significant fatty infiltration, unchanged from prior imaging.

Ten years after initial presentation, the patient returned with increased right shoulder pain after overhead injury at the gym. Her exam included a positive O’Brien’s test, and external rotation weakness. Interval EMG was normal; MRI demonstrated mild supraspinatus tendinosis, and infraspinatus atrophy with unchanged fatty infiltration (Fig. 6). The patient’s pain was hypothesized to be due to infraspinatus atrophy and overuse of other rotator cuff musculature. She elected to manage her symptoms with self-directed therapy.

## Discussion

The SSN contributes to motor control of the supraspinatus and infraspinatus and sensory innervation to the glenohumeral and AC joints [5]. Clinical symptoms include pain and diminished strength in abduction, forward flexion, and external rotation [12]. Visible infraspinatus atrophy occurs in 30%–34% [2, 13], and nonspecific pain in up to 63% of hitting shoulders in volleyball players [5, 13]. A positive cross-body adduction test and impingement signs can narrow the differential to suprascapular neuropathy [1, 13].

Initial conservative treatment is first-line, with mixed outcomes [9]. A positive Rask test after failure of non-operative treatment may necessitate surgery as demonstrated by Ferreti *et al*. Operative intervention in the early stages of suprascapular neuropathy may optimize muscle recovery in young patients [1]. Our patient underwent decompression at 18 years old, but the duration of denervation prior to presentation was unclear. With obvious infraspinatus muscle atrophy and reduced strength on examination, NCS may be warranted to maximize SSN preservation even without pain.

No case of this injury has been presented with this length of follow-up. Ferretti *et al*. found persistent infraspinatus atrophy in volleyball players managed non-operatively and operatively at 5.5 and 2 years, respectively. Similar to the operatively managed patients, our patient’s short-term outcomes post-decompression were satisfactory [1]. In contrast, Brzoska *et al*. found not only muscle bulk recovery in 70% of cases but complete restoration of infraspinatus muscle bulk in 50% of cases at 6.5 years. Other studies indicate greater capacity for muscle volume enlargement of 43.5% for complete and 47.8% for partial recovery [11].

Long-term follow-up may demonstrate less satisfactory results or even recurrence despite adequate decompression, which may be confounded by the duration of suprascapular neuropathy prior to presentation. Additionally, Brzoska *et al*.’s arthroscopic decompression was performed from the suprascapular notch to the spinoglenoid notch [11]. Our patient’s decompression was performed only at the level of the spinoglenoid notch distal into the infraspinatus.

Limitations of this case report include its retrospective nature; as a result, initial physical exam findings especially are limited to descriptions available in the medical record. Physical therapy notes were unable to be accessed. There was no muscle biopsy performed at the time of SSN decompression; our findings rely on MRI and EMG results. Future studies should aim to increase the duration of follow-up in suprascapular neuropathy and determine the role of symptom duration and decompression location on recovery of muscle bulk and function in the setting of traction injury.
